# Assessment of Preschool’s Inclusive Participation in Social Responsibility Program Under Institutional Pressure: Evidence From China

**DOI:** 10.3389/fpsyg.2022.810719

**Published:** 2022-03-09

**Authors:** Yang Lv, Chenwei Ma, Min Wu, Xiaohan Li, Xinxin Hao

**Affiliations:** ^1^College of Teachers, Chengdu University, Chengdu, China; ^2^School of Public Administration, Sichuan University, Chengdu, China

**Keywords:** early childhood education, social responsibility, preschool education, public interest kindergarten, organization psychology

## Abstract

China set the goal of expanding early childhood education (ECE) in 2018, by encouraging the development of public interest kindergartens (PIK) to provide high-quality, low-cost preschool services to the general public. This is in response to the challenges of accessibility, affordability, and accountability besetting China’s current ECE system. However, the transition toward PIK has been slow due to various complex problems, including the lackluster willingness of ECE providers to become PIK. To better understand the challenges leading to low participation, this study explores the external pressures affecting ECE providers and evaluates the external factors that influence their level of social responsibility. A stratified-random sampling questionnaire survey solicited responses from 832 ECE personnel representing 261 kindergartens from across China. Our findings suggest that institutional pressure has a positive effect on social responsibility and inclusive participation. We also found that institution visibility positively regulates the relationship between institutional pressure and social responsibility. At the same time, the level of environmental perception positively governs the relationship between social responsibility and participation willingness. Kindergartens should have certain social values, including assuming certain behaviors and participating in social activities in the spirit of social service and ensure multiple subjects’ synergetic governance.

## Introduction

Early childhood development and education (ECDE) has been recognized for its irreplaceable role in childhood development and care by the educational development community. In the United Nations Sustainable Development Goals, one of the targets is to ensure that all children have access to quality early childhood education (ECE), development, and care that prepare them for primary education (UNICEF, 2019). Early childhood is a period of development during which all young children can engage in meaningful experiential learning and social interactions (Ackah-Jnr and Udah, 2021). ECE refers to the period between the ages of three and six during which children receive various care and educational services (Hoffman et al., 2020; Wang et al., 2020). It is typically a provision, service, or practice that aims to promote social fairness and equality and reduce early disadvantages, empowering all children and enabling them to reach their full potential (Ackah-Jnr and Udah, 2021). Early infancy provides an opportunity to avoid future delays and challenges (Sarker et al., 2019).

To ensure that all children have equitable access to programs and services, inclusive early childhood education has become a significant emphasis of national governments, education systems, and institutions (Pan et al., 2020). ECDE can transform how society views early childhood education (Ho, 2010). It is motivated by the belief that inclusion and ECE are inextricably linked. Because research indicates that high-quality ECE is critical for all children, beginning inclusion in early childhood is wise and morally or ethically sound (Hu et al., 2016). In essence, early childhood inclusion is critical to the quality of early childhood programs and classrooms and a means of ensuring that all children receive an adequate start in life (Khaleel et al., 2021).

In China, total ECE providers (i.e., kindergartens) are 281,174 in 2019, while 76.96% of them are public interest kindergartens (PIK) and located in urban areas (Ministry of Education, 2020). Although urban ECDE resources are abundant, the proportion of private kindergartens willing to provide affordable educational services is somewhat limited. For example, in Chengdu, only 248 out of 917 (equivalent to 27.04%) private kindergartens are defined as PIK, who are willing to charge tuition fees within the government price range (Li et al., 2010). At the national level, the gross enrollment rate in ECE was 81.7% at the end of 2018, while the PIK coverage rate was 73.1% (Ministry of Education, 2020). These values still fall short of the target 85% enrollment rate and 80% coverage rate for 2020, as set by the government of China (Jiang et al., 2021).

The huge disparity between urban and rural (71.79% in number and 59.42%) student’s access to ECE is caused by several factors, such as high costs and difficulties entering kindergarten (Hong et al., 2015). Although China’s gross preschool enrolment rate in 2019 increased by 1.7% from the previous year. The overall imbalance in the development of preschool education has not been fundamentally changed. Due to the number, coverage, and service population requirements, preschool educational institutions have faced several challenges to become more inclusive and universal institutions (Yang and Yu, 2021). Aside from instituting changes in the national policy, strategies and measures have to be re-examined on how to stimulate and foster inclusive participation in preschool institutions. Kindergarten inclusive participation refers to the kindergarten’s willingness and active participation for the public enrollment, charging the government nominated fees for education or accepting government guidance prices, meeting the basic standards and conditions set by the government (Li et al., 2010). Inclusive participation also focuses on the participation of preschool to inclusive program like social responsibility program in China (Yang and Li, 2019).

Studies on public interest kindergartens have mainly focused on the connotation characteristics (Yong et al., 2019; Zhou et al., 2020), status quo (Winton et al., 2015; Morrison, 2018), and policy suggestions (Dahan and Senol, 2012; Józsa et al., 2018), while only few studies have dealt with the promotion mechanism and behavioral logic of pre-inclusive education. Connotation development of preschool education means the improving student’s awareness of a word so that they can use it properly even in a complex and challenging field (Yong et al., 2019; Zhou et al., 2020). The status quo enables us to innovate in order to address the needs of learners, families, and learning communities, despite established conventions and practices (Yong et al., 2019; Zhou et al., 2020). Policy suggestion mainly focuses on the policy for improving the effectiveness of preschool education (Józsa et al., 2018). Most of the relevant studies were qualitative (Escartí et al., 2010b; Zabeli and Gjelaj, 2020; Ko et al., 2021) and highly subjective (Ko et al., 2021), and very few included quantitative analyses that utilized model construction. Considering the importance of the issue and addressing research gap, this study intends to explore the external pressures affecting ECE providers and evaluates the external factors that influence their level of social responsibility. Using institutional pressure theory and social responsibility, this study hypothesizes and verifies the structural equation model of inclusive participation’s intrinsic motivation and behavioral logic in Chinese kindergartens.

This study focuses on the whole social responsibility consciousness of the industry in which preschool education institutions are located. The findings of this study can help to make a strategy for the supervision and construction of corporate social responsibility, improving the service quality, and creating a positive and harmonious atmosphere of social responsibility. Policy-makers can use the results of this study is proposing new strategies for social responsibility management of preschool education. This study can also help ECE providers and local government units to guide them in preschool education reform.

## Theoretical Framework

### Development of ECE in China

In China, early childhood education is provided for children 3–6 years old. Since its beginning in 1949, ECE in China has undergone significant changes. From 1949 to 1992, ECE focused on women’s employment and family support. At this stage, school choices were limited, and the allocation of educational resources mainly depended not on market forces but on government policies and planning (Yuan and Weian, 2019). From 1993 to 2009, the Chinese government focused on developing the market economy. It gradually deteriorated the government’s exclusive control over preschool education while encouraging the private sector and non-governmental organizations to provide ECE services. From 27,899 state-owned kindergartens in 1993, there were fewer than 10,000 in 2002 (Xiaodong and Xin, 2009). As preschool education entered a market-led growth stage, private kindergartens developed more rapidly, resulting in the accelerated surge in educational expenses and the weakening of welfare provisions in preschool education (Luo, 2011; Yuan and Weian, 2019). In 2010, ECDE gained more national attention and public interest, but its problems had become more serious. In response, the Chinese State Council has issued a series of policy changes aimed toward developing non-governmental support for public kindergartens, increasing public spending for preschools, and reshaping the concept and development orientation of PIK (Zhou et al., 2017).

### Social Responsibility and PIK

Factors that hinder the transformation of kindergartens to PIK are complex and multifaceted (Dawei, 2019). PIK’s primary goal is to provide high-quality, low-cost early childhood education services to the general public. Before the transformation of kindergartens to PIK, conceptual changes and policy modifications have to be undertaken to promote democratization and highlight the social responsibility of ECE. Though public and private kindergartens may have different development orientations, both have to serve the public interest. The ECE system needs to prioritize service and the public good over profit to encourage the transformation of kindergartens to PIK (Ping and Xinyue, 2013). It requires huge support and supervision from the government and the cooperation of the private sector.

### Theoretical Perspective

Though the issues are important, only a few studies have closely examined the social responsibility of kindergartens and the various institutional pressures and forces affecting the system within China. In recent years, the mainstream research concepts of “voluntariness principle” and “utilitarianism principle” have shifted toward macroscopic notions of “compulsory responsibility” and “voluntarism principle” (Zhang and Kecheng, 2011). As a quasi-public service product, kindergartens assume responsibilities that are compulsory and voluntary. Some scholars have pointed out that organizational behavior is easily shaped and regulated by the external environment. Social responsibility may be symbolic for kindergartens to gain organizational legitimacy (Miller and Guthrie, 2007). However, the driving mechanism of social responsibility among ECE providers remains highly controversial and contentious (Cambell and Campbell, 2007; Hess and Warren, 2008). To understand in-depth challenges, it is necessary to know what external pressures promote commitment and encourage ECE providers? Do external pressures significantly affect the behavior of kindergartens toward inclusive participation? What role does social responsibility play in the transmission process? These questions deserve further discussion.

The new institutionalism theory gradually clarified the internal mechanism of external institutional pressure and organizational behavior (Meyer and Rowan, 1977; DiMaggio and Powell, 2000). The institutional theory posits that a series of different forms of pressure, such as institutions, norms, and regulations, can further influence the behavioral choices of individuals and organizations (DiMaggio and Powell, 2000). Scott (2001) defined the institutional environment as a general term for elements, including regulatory, normative, and cultural cognition, while related activities and resources provide stability for social life. Regulatory pressure mainly refers to laws, rules, policies, and other elements promulgated by organizations with or similar to legal authority and conducive to social stability and order (Lee et al., 2017). Normative pressure is usually hidden and not easily recognizable by outsiders (Colwell and Joshi, 2013). It mainly refers to national and regional culture, values, beliefs, and behaviors and is a prescribed and obligatory dimension in social life. Cognitive pressure emanates from the understanding and recognition of the external environment of individuals and organizations, which emphasizes the importance of social identity (Lau and Li, 2018). Research has shown that institutional pressure has a significant impact on the fulfillment of corporate social responsibility (Shen et al., 2014).

Institutional theorists propose that institutional pressure causes organizations to conform to social conventions to accept and recognize legitimacy (Kostova et al., 2008). This view overlooks the role of positive response and resistance in the relationship between the organization and the environment. Luo (2011) believes that organizations will make reasonable and unique strategic choices to achieve established goals, rather than passively adapting to all institutional conditions. An institutional theory emphasizes that the more visible an organization is, the more likely its stakeholders will pay attention. The organization would be compelled to abide by accepted social norms and assume greater social responsibility and institutional expectations to obtain the legitimacy and resources needed for its survival (Yin et al., 2020). As a specific manifestation of dynamic organizational ability, environmental perception refers to an organization’s ability to detect environmental changes and identify opportunities and threats (Wang and Justin, 2009; Jiao et al., 2010). To achieve this, the organization must have an accurate understanding and grasp of social norms and standards.

**Figure 1:**
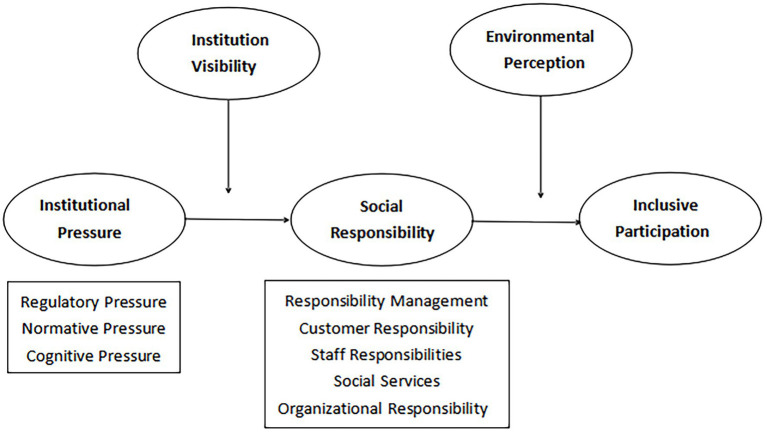
Conceptual model diagram of the study.

In China, institutional pressures on kindergartens mainly come from regulatory pressure, social norms, and cognitive pressure on organizations to promote social responsibility (Shen et al., 2014). Figure 1 presents the conceptual framework detailing the interactions between institutional pressures and external forces. The inclusive participation of kindergartens is likely a strategic response to turbulent changes in the social environment. Using a nationwide sample of kindergartens, this study aims to answer the following research questions:

- Does institutional pressure affect the social responsibility of ECE providers in China?
- Is the participation of ECE providers in social responsibility intermediary?
- Does the level of visibility among ECE providers affect institutional pressure and social responsibility?
- Does the environmental perception of ECE providers affect social responsibility and inclusive participation?

The conceptual model diagram is constructed, as shown in Figure 1.

### Research Hypotheses

#### Institutional Pressures and Social Responsibility

Cambell and Campbell (2007) argues that the three institutional pressures (i.e., regulatory, normative, and cognition) work individually or collectively in pushing enterprises to continuously improve their social performance in terms of environmental protection, product quality, employee satisfaction, and business interests. Corporate social performance embodies social responsibility influenced by different institutional pressures (Lee et al., 2017). Shen et al. (2014) found that the institutional environment, composed of rules, norms, and concepts, has a significant impact on the organizational behavior of enterprises, which they termed as institutional pressure. Wang and Justin (2009) argue that social, institutional, environmental, political, legal, and market pressures affect the actualization of corporate social responsibility (CSR). In constructing the CSR process model, Basu and Palazzo (2008) posit that the perception of an organization’s legitimacy would affect aspects of their social responsibility. Regulatory pressure on ECE providers is manifested directly through government interventions, including the formulation of new laws and regulations, modifications of existing rules and policies, and the imposition of administrative penalties. Normative pressure mainly refers to the peer benchmarking role of preschool institutions and the impact of public expectations on the social responsibility of ECE providers (Khamzina et al., 2021). In comparison, cognitive pressure mainly refers to the understanding and consideration of social responsibility by the ECE providers themselves. ECE exists in a particular social environment, which affects many stakeholders, and the performance of the social responsibilities of ECE providers can be influenced by institutional pressure. Accordingly, we propose the following hypotheses:

*H1*: Institutional pressure positively influences social responsibility.

*H1a*: Regulatory pressure positively influences social responsibility.

*H1b*: Normative pressure positively influences social responsibility.

*H1c*: Cognitive pressure positively influences social responsibility.

#### Institutional Pressures and Inclusive Participation

According to institutional theory, the organizational choice is limited by various external pressures because the environment is collective and interrelated (DiMaggio and Powell, 2000). Institutional pressure can have a considerable impact on organizational behavior. Under institutional pressure, enterprises conform to policy norms and make decisions that are similar to common industry practices to obtain the recognition and support of stakeholders (Meyer and Rowan, 1977). Chen et al. (2015) found that institutional factors, such as laws, rules, and beliefs, can constrain the structure and behavior of enterprises. Instead of making decisions independently, enterprises must respond to external needs and expectations to improve their survival rate, which means conforming their choices to follow social norms (Rotjanakorn et al., 2020). Colwell and Joshi (2013) found that institutional pressure positively affects the bidirectional innovation willingness of small and medium enterprises (SMEs). Wang et al. (2020) developed an empirical model on the impact of institutional pressure on environmental management strategies and practices. They found that imitative and normative force had a significant impact on environmental management and practices. Zhao and Jing (2019) found that the three dimensions of institutional pressure had a significant positive effect on the pro-environment behavior of SMEs. In this study, we explore and investigate the inclusive participation behavior of ECE providers based on the institutional theory. We propose the following hypotheses:

*H2*:Institutional pressure positively influences inclusive participation.

*H2a*: Regulatory pressure positively influences inclusive participation.

*H2b*: Normative pressure positively influences inclusive participation.

*H2c*: Cognitive pressure positively influences inclusive participation.

#### Social Responsibility and Inclusive Participation

Abendroth and Heyman (2013) used social and market norms to explain the utility incentive of behavioral intention to participate. Market norms belong to economic factors, which involve the time cost and the degree of obtaining participants’ benefits. Social norms usually govern behaviors not measured by economic parameters but by actions and activities contributing to social rules and obtaining the psychological sense of achievement. Examples include altruism, politeness, and reciprocity (Heyman and Ariely, 2004). From the perspective of the dialogue concept, Bruning et al. (2008) found that the expectations of the public and social organizations to acquire social values would promote participation in social affairs. Using an empirical approach based on a system dynamics model, Gold et al. (2018) concluded that multiple participants in society encourage participation in social governance through non-material utilities, such as improving public relations and cultivating a favorable reputation. Since China has entered the market economy system, social organizations must pursue their economic interests and consider their social responsibilities when participating in social governance (Han et al., 2016). ECE providers can pursue non-monetary benefits in preschool education and assume social responsibilities to obtain social rewards. Accordingly, we propose the following hypotheses:

*H3*: Social responsibility positively influences inclusive participation.

*H4*: Social responsibility plays a mediating role between institutional pressure and inclusive participation.

#### Institutional Visibility Regulates the Relationship Between Institutional Pressure and Social Responsibility

Visibility refers to the degree to which an enterprise attracts the attention of various stakeholders, such as the public, regulators, and the media, and is an important feature of strategic corporate social responsibility (Hadi and Udin, 2021). The visibility in engaging in corporate social responsibility activities can enhance an enterprise’s reputation and provide the ability to maintain competitive advantages. Rotjanakorn et al. (2020) argue that enterprises with high visibility can easily attract the government’s attention, leading to more government support and interventions. Chiu and Sharfman (2011) found that visibility could prompt enterprises to adjust their behaviors and respond positively to stakeholder pressure. They also believe that managers would actively fulfill their social responsibilities in response to social expectations and legal pressure and try to become corporate citizens of the society. Capriotti (2009) suggests that public visibility can help explicitly highlight an enterprise’s CSR. Saiia et al. (2003) explored stakeholders’ pressures on CSR and analyzed how visibility can motivate large enterprises to increase their charitable donations. For ECE providers, the main pressure emanates largely from government regulations, social norms, and the cognitive strain to encourage social responsibility. Accordingly, the following hypotheses are proposed in this study:

*H5*:Institution visibility positively affects the relationship between institutional pressure and social responsibility.

*H5a*: Institution visibility positively affects the relationship between regulatory pressure and social responsibility.

*H5b*: Institution visibility positively affects the relationship between normative pressure and social responsibility.

*H5c*: Institution visibility positively affects the relationship between cognitive pressure and social responsibility.

#### Environmental Perception Regulates the Relationship Between Social Responsibility and Inclusive Participation

Environmental perception means that an enterprise can fully recognize the changes and development trends of the environment (Ismail et al., 2014). Prahalad and Hamel (2009) suggest that enterprises should have a deep understanding of their industries’ development and operation rules while capturing possible changing trends. Li (2006) conducted multiple case studies and found that adaptive behaviors of enterprises to environmental changes initially emanate from their capacity to fully understand the environment, which includes the ability to analyze the impact of environmental changes on the market. Environmental awareness can encourage enterprises to make high-quality decisions and promote resource efficiency more quickly (Teece et al., 2016). This effect of environmental awareness on businesses can also have the same impact on ECE providers. Inclusive participation of kindergartens can result in strategic responses to the turbulent changes in the social environment. Accordingly, the following hypotheses are proposed in this study:

*H6*:Environmental perception positively affects the relationship between social responsibility and inclusive participation.

*H6a*: Environmental perception positively affects the relationship between responsibility management and inclusive participation.

*H6b*: Environmental perception positively affects the relationship between customer responsibility and inclusive participation.

*H6c*: Environmental perception positively affects the relationship between staff responsibility and inclusive participation.

*H6d*: Environmental perception positively affects the relationship between social services and inclusive participation.

*H6e*: Environmental perception positively affects the relationship between organizational responsibility and inclusive participation.

## Research Methodology

### Research Settings

The primary research object in this study is Chinese ECE providers. With the rapid development of China’s economy and society, preschool education has received significant attention from the government and the people. Along with this heightened interest, China’s current “3A” problems (i.e., Accessibility, Affordability, and Accountability) in ECE providers have become highly pronounced and require considerable strengthening of the country’s PIK. Preschool education in most developed countries has also long been oriented to serve the public interests. In other countries, such as China, ECE requires significant policy overhaul and long-term development strategies. PIK development is a key focus in the United Nations Sustainable Development Goals on Quality Education. It aims to ensure that children have access to quality early childhood development, care, and preschool education by 2030 to prepare them for primary education.

### Measurement of Variables

Institutional pressure was measured by measuring regulatory, normative, and cognitive pressure. Similar methodology was used by Brammer et al. (2012) and Lee et al. (2017). Social responsibility was assessed by measuring responsibility management (Wigmore-Álvarez et al., 2020), customer responsibility (Rotjanakorn et al., 2020), staff responsibility (Zhou et al., 2017), social services (Lau and Li, 2018), and organizational responsibility (Zhou et al., 2020). Similarly, institution visibility (Tometten et al., 2021), environmental perception (Hoffman et al., 2020), and inclusive participation (Schwab et al., 2021) were evaluated by respective indicators shown in Table 1. The questionnaire design consists of two parts. The first section solicits the basic information of the respondents and ECE providers, while the second part measures different parameters and willingness variables that may affect inclusive participation. Five latent variables were used in the study, including institutional pressure, social responsibility, institutional visibility, environmental perception, and inclusive participation. A five-point Likert scale was used for each variable to indicate the respondent’s level of agreement: from complete disagreement (1) to complete agreement (5). The choice of the latent and observed variables used in the study is based on the findings and recommendations from previous studies and was modified based on the context of preschool education, literature reviews, and expert consultation. Common method variation was used to check suitability of the dataset. Similarly, confirmatory factor analysis was then conducted to evaluate the suitability of the model. Descriptive statistics was done to obtain the demographic features of the respondent. Hierarchical regression was applied to show impact of regulatory, normative, and cognitive pressure on social responsibility. Finally, the bias-corrected bootstrapping test helps to analyze the mediating effect of social responsibility between internal pressures and inclusive participation. Table 1 provides a summary of the variables used and the associated references.

**Table 1:** Selection of variables and expected relationship with preschool’s inclusive participation.

Variables	Indicators	Relationships with preschool’s inclusive participation	Sources
Institutional Pressure	Regulatory Pressure	Governments have strict punishment measures for violations of social responsibility (such as failing teaching quality, accepting bribes and non-transparency of information)	Clemens and Douglas, 2005; Farjoun and Starbuck, 2007; Escartí et al., 2010a; Luo, 2011; Colwell and Joshi, 2013; Yin, 2017
		Governments protect the interests of kindergartens through measures such as reporting and strict law enforcement	
		Governments publicize the concept of social responsibility in kindergartens through various forms (such as WeChat official accounts, website forums, and news reports)	
		The state quickly responds to the public’s violation of social responsibility behavior (such as suspension of trading, rectification)	
	Normative Pressure	The kindergarten will learn about the social responsibility concept of market subjects from the industry or vocational association (relevant departments such as the Education Bureau)	Husted and Allen, 2006; Basu and Palazzo, 2008; Colwell and Joshi, 2013; Lee et al., 2017; Shafi et al., 2020; Zhou et al., 2020
		The kindergarten’s philosophy of social responsibility (such as children’s food, safety, and health) is highly appreciated by the local public	
		The kindergarten principals and teachers accept social responsibility education has a strong influence on the kindergarten	
	Cognition Pressure	Peer kindergarten has expanded its popularity because of its better performance of social responsibilities	Clemens and Douglas, 2005; Kostova et al., 2008; Mueller et al., 2012; Yin, 2017; Lau and Li, 2018
		The kindergarten pays close attention to the strategies and actions of peers in public relations	
		A kindergarten that does an excellent job of social responsibility will have a good reputation among its peers	
		Peer kindergartens have gradually strengthened their social responsibility system construction	
Social Responsibility	Responsibility Management	The kindergarten has a specific strategic plan for social responsibility	Tate et al., 2010; Luo, 2011; Merchant et al., 2012; Wigmore-Álvarez et al., 2020
		The kindergarten regularly publishes social responsibility reports	
		The kindergarten can effectively use government policy loans and subsidies	
	Customer Responsibility	The kindergarten has a comprehensive safety accident response mechanism for children	Prahalad and Hamel, 2009; Dahan and Senol, 2012; Merchant et al., 2012; Yin, 2017; Simionescu and Dumitrescu, 2018; Rotjanakorn et al., 2020; Ko et al., 2021
		The kindergarten has a comprehensive video surveillance system	
		The kindergarten has a unique teaching philosophy and a detailed teaching plan	
		The kindergarten has high teaching quality and parent satisfaction	
		The kindergarten has a parent and student personal information security management mechanism	
		The kindergarten has an effective student and parent tracking service and complaint management system	
	Staff Responsibility	The kindergarten complies with the rights and interests of employees stipulated by laws, regulations, or collective agreements (e.g., wages, welfare, social security)	Zhou et al., 2017; Wigmore-Álvarez et al., 2020; Zabeli and Gjelaj, 2020; Ackah-Jnr and Udah, 2021
		The kindergarten offers equal opportunities for career development	
		The kindergarten provides education and training for employees	
	Social Services	The kindergarten serves special needs, such as children with disabilities	Kelley et al., 2008; Merchant et al., 2012; Ismail et al., 2014; Lau and Li, 2018
		The kindergarten actively organizes and promotes social service activities in the community	
		The kindergarten has a fair and inclusive pricing policy	
	Organizational Responsibility	The kindergarten complies with national laws and regulations/legal operations	Kelley et al., 2008; Merchant et al., 2012; Ivy et al., 2018; Zhou et al., 2020
		The kindergarten has a good reputation and a positive image	
		The kindergarten has drinking water, sanitation, teaching facilities, construction, and other safety and quality assurance	
Institution visibility		The kindergarten’s education service level and development status are always concerned and supervised by shareholders and investors	Clemens and Douglas, 2005; Capriotti, 2009; Chiu and Sharfman, 2011; Dahan and Senol, 2012; Wigmore-Álvarez et al., 2020; Zabeli and Gjelaj, 2020; Tometten et al., 2021
		The kindergarten’s education service level and development condition are always concerned and supervised by the trade union	
		The kindergarten’s education service level and development status are always concerned and supervised by the government regulatory agencies	
		The kindergarten’s education service level and development status are always concerned and supervised by the media, community public, and industry organizations	
		This kindergarten’s education service level and development status are always concerned and supervised by peers and parents of students	
Environmental perception		The kindergarten has a good understanding of the development and operation rules in the field of preschool education	Colwell and Joshi, 2013; Ismail et al., 2014; Shafi et al., 2019; Hoffman et al., 2020; Wigmore-Álvarez et al., 2020; Schwab et al., 2021
		The kindergarten is fully aware of the changes and trends in the environment and plans to respond well in advance	
		The kindergarten can frequently communicate with peers, students, and parents to get useful information from them in time	
		The kindergarten can detect changes in industry trends before most competitors	
Inclusive participation		We are willing to participate in inclusive preschool education public services actively	de Leeuw et al., 2019; Zabeli and Gjelaj, 2020; Ackah-Jnr and Udah, 2021; Schwab et al., 2021
		We are willing to increase investment in inclusive preschool public services	
		We are willing to participate in inclusive preschool public services as long-term work	

Table 1. Selection of variables and expected relationship with preschool’s inclusive participation.

### Data Collection

By following convenience sampling approach, a total of 1,050 questionnaires were distributed to 400 ECE providers in China’s 31 provincial-level administrative divisions, of which 1,050 questionnaires were recovered, and 832 were valid. The valid samples cover 261 kindergartens, which come from 27 provinces and 103 cities. Among the valid samples, 46.1% are from economically developed regions, where kindergartens are more representative and typical. To ensure the quality and accuracy of results, the samples were taken from people top the management level according to inclusion criteria. Each questionnaire takes around 30 min to answer all questions. The descriptive statistics of the valid samples are summarized in Table 2. The majority of the respondents are female, between 25–35 years old, bachelor’s degree holders, have a length of service between 5–10 years, and come from basic management or higher level. A majority of the kindergartens sampled have been established for at least 5 years and offer five classes or more. The number of public and private kindergartens sampled is almost equal, and a considerable majority is classified as public interest kindergarten (PIK).

**Table 2:** Sample descriptive statistics.

Demographic variables	Group	Frequency	Percentage
Gender	Male	34	4.1%
	Female	798	95.9%
Age	Under 25 years old	8	1%
	25–35 years old	512	61.4%
	35–45 years old	260	31.3%
	45–55 years old	52	6.3%
	Over 55 years old	0	0%
Education	Junior high school	0	0%
	Senior high school	10	1.2%
	College degree	278	33.4%
	Bachelor degree	536	64.4%
	Master degree	8	1%
Length of service	Under 5 years	104	12.5%
	5–10 years	444	53.4%
	10–15 years	200	24%
	Over 15 years	84	10.1%
Title and rank	Senior management	20	2.4%
	Middle management	178	21.4%
	Basic management	250	30%
	General staff	384	46.2%
Years of establishment (Number of kindergartens)	Within one year	1	0.4%
	1–5 years	23	8.8%
	5–10 years	108	41.4%
	Over 10 years	129	49.4%
Number of classes (Number of kindergartens)	Under 5 classes	6	2.3%
	5–10 classes	101	38.7%
	Over 10 classes	154	59%
Nature of the Kindergartens (Number of kindergartens)	Public kindergartens	135	51.7%
	Private kindergartens	126	48.3%
Whether it is public interest kindergarten (Number of kindergartens)	Yes	228	87.3%
	No	33	12.7%

### Statistical Analysis

After collecting the questionnaires, the data were recorded according to the requirements of social statistical analysis. SPSS22.0 and Amos24.0 software were used to analyze the sample data. Before conducting hypothesis testing, Harman’s single-factor detection method was used to test for the common method variation (CMV). This CMV approach is simple and easy to use. Since the scales used in this study are all recognized, exploratory factor analysis and confirmatory factor analysis were conducted to determine whether the indexes adopted have structural validity.

### Ethical Approval

All ethical guidelines and standards of the research were followed in the study. Ethical approval was obtained from the ethics review committee of Sichuan University, Chengdu, China. Besides, verbal approval was obtained from each interviewee before an interview. This study also maintained the confidentiality of the interviewee.

## Results

### Common Method Variation

Using Harman’s single-factor detection test, the existence of CMV was examined in the survey data. If the variance explained by the first common factor reaches 50% or more, it would suggest that there is significant CMV present in the dataset. The exploratory factor analysis found that the first principal component variance interpretation rate was 27.33% in this study. Since the calculated value is less than 50%, this indicates that the dataset does not contain serious CMV and that the accuracy of the results is not considerably affected (Chang et al., 2010).

### Model Fitness

The results of the exploratory factor analysis showed KMO = 0.900 (df = 703, *p* = 0.000). Confirmatory factor analysis was then conducted to evaluate the suitability of the model. The best model can be determined by comparing multi-factor models. As shown in Table 3, the fitness of the five-factor model is significantly better than other models, and the values are as: *X*^2^/DF = 1.200, CFI = 0.991, NFI = 0.950, TLI = 0.990, IFI = 0.991, and RMSEA = 0.022. The five-factor model passes the confirmative factor analysis (Table 3). Similar analysis was done by Ren and Zhang (2020) for Chinese preschool situated in Hong Kong.

**Table 3:** Model fit degree.

Model	*X*^2^/df	NFI	TLI	IFI	CFI	RMSEA
Single-factor model	13.987	0.461	0.416	0.480	0.477	0.177
Two-factor model	10.372	0.541	0.510	0.567	0.564	0.150
Three-factor model	5.811	0.746	0.748	0.780	0.779	0.108
Four-factor model	4.970	0.787	0.792	0.822	0.821	0.098
Five-factor model	1.200	0.950	0.990	0.991	0.991	0.022

NFI, Normed Fit Index; TLI, Tucker Lewis index; IFI, Incremental Fit Index; CFI, Comparative Fit Index; and RMSEA, Root Mean Square Error of Approximation.

### Descriptive Statistics and Reliability of Variables

Table 4 presents the mean value, standard deviation, correlation matrix, and reliability and validity of variables used in the study. *α* > 0.7, AVE > 0.5 indicates that each variable has good reliability and convergence validity (Purnomo, 2017). Since the square root of AVE value for each variable is greater than the correlation coefficient between the variable and the other parameters, the results suggest all the variables have good discriminant validity. Similar analysis have been done by Hong et al. (2020) for their study.

**Table 4:** Descriptive statistics, reliability, and validity of variables.

S. No.	Variables	*N*	Mean	*SE*	Convergent validity		Distinction validity						
					*α*	AVE	1	2	3	4	5	6	7
1.	Regulatory Pressure	416	3.809	0.878	0.837	0.569	1						
2.	Normative pressure	416	3.859	0.918	0.806	0.584	0.458^**^	1					
3.	Cognitive Pressure	416	3.848	0.867	0.856	0.601	0.229^**^	0.405^**^	1				
4.	Social responsibility	416	3.671	0.753	0.926	0.508	0.453^**^	0.599^**^	0.456^**^	1			
5.	Institution visibility	416	3.840	0.886	0.889	0.620	0.294^**^	0.324^**^	0.295^**^	0.313^**^	1		
6.	Environment perception	416	3.740	0.869	0.869	0.633	0.050	0.096	0.122^*^	0.194^**^	0.078^**^	1	
7.	Inclusive participation	416	4.023	0.811	0.791	0.561	0.526^**^	0.651^**^	0.491^**^	0.655^**^	0.341^**^	0.243^**^	1

### Main Effect and Regulating Effect Test

To ensure the accuracy of the research results, the age, size, and nature of the ECE provider were included in the hierarchical regression as control variables and whether the ECE provider is a PIK. For Model 2, additional independent variables were added to Model 1, and the summary of results is presented in Table 5. The regression results show that regulatory pressure (*β* = 0.174, *p* < 0.001), normative pressure (*β* = 0.259, *p* < 0.001), and cognitive pressure (*β* = 0.183, *p* < 0.001) are all positively affected by social responsibility. Model 3 is based on Model 2 that incorporates regulatory variables. The regression results from Model 3 show that institution visibility (*β* = 0.117, *p* < 0.01), institution visibility^*^regulatory pressure (*β* = 0.079, *p* < 0.05), institution visibility^*^normative pressure (*β* = 0.159, *p* < 0.01), and institution visibility^*^cognitive pressure (*β* = 0.143, *p* < 0.01) have significant positive correlation with social responsibility. The results also indicate that the positive impact of regulatory, normative, and cognitive pressure on social responsibility performance will be strengthened, and the moderating effect is established in the case of high organizational visibility.

**Table 5:** Regression test of main effects and regulating effects.

Variables	Social responsibility			Inclusive participation			
	1	2	3	4	5	6	7
Control Variables	Years of establishment	0.173^**^ (0.058)	0.135^**^ (0.049)	0.152^**^ (0.045)	0.229^**^ (0.058)	0.186^**^ (0.046)	0.130^**^ (0.044)	0.125^**^ (0.041)
	Number of classes	0.180^*^ (0.073)	0.190^**^ (0.060)	0.222^**^ (0.056)	0.278^**^ (0.074)	0.289^**^ (0.060)	0.211^**^ (0.057)	0.186^**^ (0.049)
	Nature of the Kindergartens	0.368^**^ (0.075)	0.233^**^ (0.063)	0.167^**^ (0.059)	0.435^**^ (0.074)	0.277^**^ (0.062)	0.181^**^ (0.060)	0.150^**^ (0.052)
	Whether it is public interest kindergarten	0.062 (0.106)	0.143 (0.090)	0.228^**^ (0.081)	−0.030 (0.113)	0.071 (0.099)	0.013 (0.090)	0.018^***^ (0.076)
	Regulatory pressure		0.174^***^ (0.036)	0.174^***^ (0.032)		0.234^***^ (0.039)	0.162^***^ (0.039)	0.149^***^ (0.033)
	Normative pressure		0.259^***^ (0.036)	0.289^***^ (0.034)		0.277^***^ (0.038)	0.170^***^ (0.034)	0.180^***^ (0.031)
	Cognitive pressure		0.183^***^ (0.039)	0.205^***^ (0.035)		0.219^***^ (0.037)	0.143^***^ (0.036)	0.139^***^ (0.031)
Intermediary Variables	Social responsibility						0.412^***^ (0.047)	0.433^***^ (0.040)
Regulatory Variables	Institution visibility			0.117^**^ (0.035)				
	Institution visibility ^*^ Regulatory pressure			0.079^*^ (0.038)				
	Institution visibility ^*^ Normative pressure			0.159^**^ (0.039)				
	Institution visibility^*^ Cognitive pressure			0.143^**^ (0.036)				
	Environmental perception							0.138^***^ (0.030)
	Environmental perception ^*^Social responsibility							0.328^**^ (0.038)
	Environmental perception ^*^Responsibility management							0.100^**^ (0.039)
	Environmental perception ^*^Customer responsibility							0.126^***^ (0.041)
	Environmental perception^*^ Staff responsibility							−0.016 (0.034)
	Environmental perception ^*^ Social services							0.099^*^ (0.042)
	Environmental perception ^*^Organizational responsibility							0.018 (0.034)
Constant term		1.961^**^ (0.278)	−0.196^**^ (0.271)	−1.057^**^ (0.240)	1.924^**^ (0.301)	−0.643^*^ (0.313)	−0.562 (0.300)	−1.045^**^ (0.253)
*R* ^2^		0.090	0.374	0.487	0.132	0.468	0.559	0.655
△*R*^2^		0.081	0.363	0.473	0.124	0.459	0.551	0.643

* *p* ≤ 0.05;

** *p* ≤ 0.01;

*** *p* < 0.001.

Additional independent variables were added to Model 5. The results show regulatory pressure (*β* = 0.234, *p* < 0.001), normative pressure (*β* = 0.277, *p* < 0.001), and cognitive pressure (*β* = 0.217, *p* < 0.001) positively influence inclusive participation. In Model 6, the mediating variable social responsibility was added to the model and used as an independent variable. The results show that social responsibility (*β* = 0.412, *p* < 0.001) is positively affected by inclusive participation. Model 7 is based on Model 6 and includes regulatory variables. The regression results from Model 7 show environmental perception (*β* = 0.138, *p* < 0.001), environmental perception^*^social responsibility (*β* = 0.328, *p* < 0.01), environmental perception^*^responsibility management (*β* = 0.100, *p* < 0.01), environmental perception^*^customer responsibility (*β* = 0.126, *p* < 0.001), and environmental perception^*^social services (*β* = 0.099, *p* < 0.05) positively affect inclusive participation.

**Figure 2:**
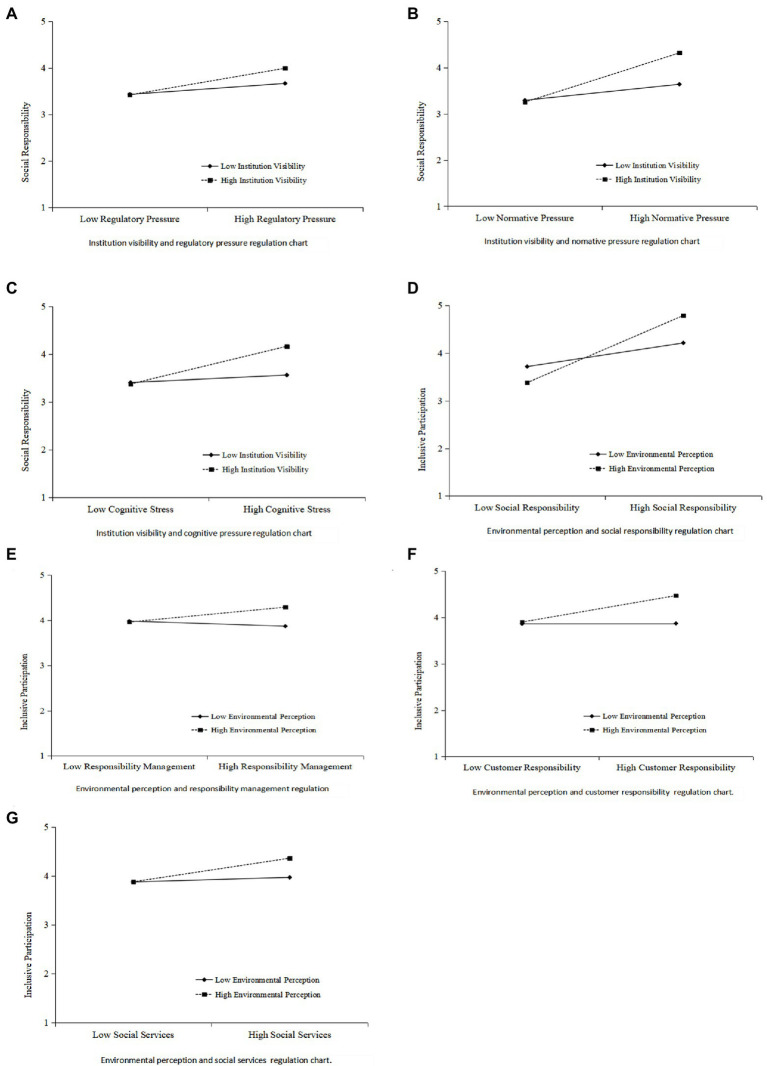
Regulation effect diagrams [**(A)** Institution visibility and regularity pressure; **(B)** Institution visibility and normative pressure; **(C)** Institution visibility and cognitive pressure; **(D)** Environmental perception and social responsibility; **(E)** Environmental perception and responsibility management; **(F)** Environmental perception and customer responsibility; and **(G)** Environmental perception and social services].

In contrast, environmental perception^*^staff responsibility (*β* = 0.016, *p* > 0.05) and environmental perception^*^organizational responsibility (*β* = 0.018, *p* > 0.05) did not have significant effect on inclusive participation. This suggests that the positive influence of social responsibility, responsibility management, customer responsibility, and social service on inclusive participation should be strengthened with acute environmental perception ability (Figure 2).

To further demonstrate the regulation effect of the regulatory variables on institution visibility and environmental perception, the slope test was conducted with high and low groups. The results show that the high-level group has a more substantial regulation effect than the low-level group. The slope lines of the two groups intersect, which means that the regulating impact exists.

### Mediating Effect Test

The bias-corrected bootstrapping test was used to analyze the mediating effect of social responsibility between internal pressures and inclusive participation. The summary of the results is presented in Table 6. The modeling paths are as follows: regulatory pressure → social responsibility → inclusive participation, normative pressure → social responsibility → inclusive participation, and cognitive pressure → social responsibility → inclusive participation. After adding social responsibility as the mediating variable, the influencing coefficients decreased in value from 0.234 to 0.197 for regulatory pressure, 0.277–0.227 for normative pressure, and 0.219–0.200 for cognitive pressure. The values of *p* were significant in all three parameters, indicating that there was partial mediation. In addition, four dimensions of social responsibility, i.e., customer responsibility, staff responsibility, social service, and organizational responsibility, were found to have significant intermediary roles, while responsibility management did not show any significant role.

**Table 6:** Mediation effects of social responsibility dimensions.

Path	Mediation Effect	Bias Corrected (95%)		Dimensions of social responsibility				
		LLCI	ULCI	Responsibility Management	Customer Responsibility	Staff Responsibility	Social Services	Organization Responsibility
Regulatory pressure → Social responsibility → Inclusive participation	0.197^***^ (0.032)	0.140	0.266	0.015 (0.011)	0.065^***^ (0.017)	0.037^**^ (0.013)	0.042^**^ (0.015)	0.038^*^ (0.015)
Normative pressure→ Social responsibility→ Inclusive participation	0.227^***^ (0.026)	0.175	0.279	0.018 (0.014)	0.076^***^ (0.020)	0.043^***^ (0.013)	0.049^**^ (0.018)	0.042^*^ (0.018)
Cognitive pressure → Social responsibility → Inclusive participation	0.200^***^ (0.031)	0.147	0.266	0.015 (0.012)	0.067^***^ (0.017)	0.038^**^ (0.013)	0.042^**^ (0.014)	0.039^*^ (0.017)

LLCI, Lower level of confidence interval, ULCI, UPPER level of confidence interval.

* *p* ≤ 0.05;

** *p* ≤ 0.01;

*** *p* < 0.001.

## Discussion

The study’s main purpose is to explore social responsibility conditions and dynamic mechanisms among ECE providers in China. Based on institutional theory, we evaluated the effects of different institutional pressure factors on social responsibility and inclusive participation in kindergartens and the regulating role of institution visibility and environmental perception. The study’s findings can help better understand the emergence and development of public interest kindergartens and can be used as a reference for follow-up studies.

### Influence of Institutional Pressure and Social Responsibility on Inclusive Participation

This study reveals that regulatory pressure, normative pressure, and cognitive pressure significantly affect ECE providers’ inclusive participation. Among these three, the influence of normative pressure is highest. According to the institutional theory, the institutional environment is collective and interrelated. The choices made by any organization are subject to various institutional pressure (Colwell and Joshi, 2013). The existing research and institutional proof pressure of the three dimensions of organizational decision making and behavior have a significant role (Merchant et al., 2012). Different institutional pressure has a significant positive effect on the decision-making behavior of ECE providers. Similarly, Lee et al. (2017) argue that institutional pressure is necessary for getting better performance for CSR practices.

In addition, the social responsibility of ECE providers has a significant positive impact on inclusive participation. Compared with institutional pressure, social responsibility has a stronger effect on inclusive participation. Colwell and Joshi (2013) reveals that institutional pressure has a positive effect on an institution to behave more responsibly to society. With China’s continued liberalization policy and economic reforms, social organizations are mandated to pursue their economic interests and consider their social responsibilities (Liu et al., 2021). Kindergartens should have certain social values, including assuming certain behaviors and participating in social activities in the spirit of social service. The findings are in line with a study of Yin (2017). Yin (2017) conducted a study on institutional drivers of CSR and argues that institutional pressures influence the likelihood of an organization to play socially responsible roles.

### The Partial Mediation Effect of Social Responsibility Between Institutional Pressure and Kindergarten Inclusive Participation

The influence of institutional pressures on the inclusive participation of kindergartens is affected by the mediating role of social responsibility. Numerous studies have pointed out that organizations may exhibit better social performance (Cambell and Campbell, 2007). Institutional pressure embodied in the formal system’s stress constraint, such as the government formulates or laws, regulations, rules, policies, and administrative punishment to force organizations to conform to the social expectation of social responsibility action measures. It is also reflected in the easing of informal normative and cognitive pressures, such as partners in the communication process through the maintenance of their rights. It can exert pressure on organizations to carry out the social responsibility of action and public opinion, ethics, and value standard constraints. The study results show that social responsibility plays an important role in the response of ECE providers to institutional pressure. Therefore, it is necessary to fully and accurately recognize the characteristics of social responsibility to have a competitive advantage in the complex and changeable market environment. In addition, the results show that the responsibility management aspect of social responsibility did not play an intermediary role between institutional pressure and inclusive participation. The preschool education development level may cause China’s preschool education development level, which is still in the early stages of reform. The relevant management institutions have not yet fully matured, particularly the management component related to social responsibility.

### Regulation of Institutional Visibility and Environmental Perception

Institutional visibility and environmental perception of kindergartens were found to moderate the fulfillment of social responsibilities and inclusive participation positively. Institutional visibility enables organizations to adjust their behavior and actively respond to stakeholders’ pressure and change public attitude through advertising and public relations activities (Bruning et al., 2008). Likewise, institutional visibility can modify the reputation and image of ECE providers. The public perception toward institutions can significantly be improved through media exposure of activities related to social responsibility. In addition, kindergartens can help improve their educational services and competitiveness by having a better understanding of the social environment that would allow for higher organizational consistency and adaptability.

The results also found that environmental perception did have a moderating effect on staff responsibility and organizational responsibility, which is contrary to the findings in more developed countries (Farjoun and Starbuck, 2007). Environmental awareness of kindergartens is necessary to make strategic adjustments need to invest more resources. These resources will bring extra burden and pressure and resource mobilization ability level. It is difficult to support the unpredictable environmental changes. The weak foundation of kindergarten is unable to make a change and counteract the effects of environmental change.

## Conclusion and Recommendations

This study has identified five key variables for measuring the inclusive participation of kindergarten in social responsibility programs under institutional pressure. These variables are institutional pressure, social responsibility, institutional visibility, environmental perception, and inclusive participation. This study has revealed a significant relationship among them. More specifically, regulatory pressure, normative pressure, and cognitive pressure significantly affect ECE providers’ inclusive participation. The influence of institutional pressures on the inclusive participation of kindergartens is affected by the mediating role of social responsibility. Institutional visibility and environmental perception of kindergartens positively moderate the fulfillment of social responsibilities and inclusive participation. This suggests that the positive influence of social responsibility, responsibility management, customer responsibility, and social service on inclusive participation will be strengthened with acute environmental perception ability.

The main theoretical contributions of the study are as follows:

First, the study focuses on the influence of social responsibility performance of ECE providers on inclusive participation under different institutional pressures. It proposes the variables of institution visibility and environmental perception from the perspective of organizational characteristics and organizational cognition, with good reliability and validity. Although many scholars have used relevant variables in their studies, the dimensions and contents of relevant variables have not been consistent, and most adopt substitution variables (Clemens and Douglas, 2005). This study adds to the growing literature on institutional pressure theory and social responsibility and provides an effective measurement approach in evaluating influencing factors on preschool education.

Second, this study explores the formation mechanism of institutional pressure on kindergarten social responsibility performance and inclusive participation. The study results highlight the effect and interaction of social responsibility and institutional pressure among ECE providers, underscore the regulating effect of institution visibility, and verify the regulating influence of inclusive participation in the context of environmental perception. This study explains how the institution’s visibility and environmental perception produce varying responses under different institutional pressures from theoretical and empirical perspectives. The approach used in the study improves the single model and develops an innovative theoretical framework using organizational characteristics and organizational cognition theories.

Finally, previous studies on institutional pressure as a pre-factor of social responsibility have mainly focused on enterprises and rarely on educational, particularly preschool education. The study links institutional pressure with ECE’s social responsibility and explores the relationship between them. This can be used as a reference when analyzing and contrasting how institutions and organizations from different industries respond to institutional pressure.

### Policy Implications

These findings of this study are of great practical significance to the ECE system. The results can be used to better deal with different institutional pressure environments, develop strategies for long-term survival and development, and promote inclusive participation.

For the government, policies and regulations have to be improved, and more incentive mechanisms and punishment measures have to be adapted to encourage social responsibility among ECE providers. One concrete way to promote social responsibility is to build an evaluation index system to measure social responsibility and inclusiveness among kindergartens. In the social aspect, the charitable and public welfare aspects of ECE should be highlighted. Kindergartens and social organizations should be encouraged to jointly implement collaborative strategies to realize and achieve social responsibility goals. Among ECE providers, kindergartens should be encouraged to set-up a system of internal governance that would help each institution actively partake in their social responsibilities and establish a benchmarking image.

More attention should be paid to the influence of the institution’s visibility of social responsibility behaviors on regulating institutional pressure on social responsibility. ECE providers can enhance their reputation by publicizing their social responsibility activities in media and highlighting their social responsibility behaviors. To enhance their influence, ECE providers can combine social responsibility activities with institutional development strategies. At the same time, the media can draw the attention of the government, society, and the public by highlighting stories that showcase social responsibility activities and conducting assessment reports of various industries regarding their level of community engagement and social responsibility performance.

Moreover, kindergartens need to perceive opportunities and build an organizational structure that can adapt to the complex changing environment. ECE providers need to instill the ability to tolerate conflicts and integrate multiple tasks and resources. Developing the ability to adjust internal resources to adapt to a particular set-up or environment can help ECE providers gain long-term advantages in the dynamic and complex environment.

### Limitations

This study has several limitations. First, in the aspect of institutional pressure, inclusive participation is affected by social responsibility, institution visibility, and environmental perception, which has a considerable temporal effect. Future studies can use intertemporal (time-series) data to analyze and explore the causal relationship between these research variables. Second, this study developed a structural equation model that statistically analyzed the role path of inclusive participation. However, certain details and variables may have been overlooked, which could affect the findings. Future studies can go deeper into the subject matter.

## Data Availability Statement

The raw data supporting the conclusions of this article will be made available by the authors, without undue reservation.

## Author Contributions

YL have designed the research plan, collected the data, analyzed the data, and wrote the manuscript. YL, CM, MW, XL, and XH analyzed the data and revised the manuscript. All authors have checked the final version of the manuscript and approved for publication.

## Funding

The article is funded by the National Social Science Fund of China Education Science Western Region Project (project approval no. XJA190284) entitled “The Behavior Logic and Realization Mechanism of Multiple Subjects’ Synergetic Governance on Vocational Education.”

## Conflict of Interest

The authors declare that the research was conducted in the absence of any commercial or financial relationships that could be construed as a potential conflict of interest.

## Publisher’s Note

All claims expressed in this article are solely those of the authors and do not necessarily represent those of their affiliated organizations, or those of the publisher, the editors and the reviewers. Any product that may be evaluated in this article, or claim that may be made by its manufacturer, is not guaranteed or endorsed by the publisher.
